# Dimensioning Cuboid and Cylindrical Objects Using Only Noisy and Partially Observed Time-of-Flight Data

**DOI:** 10.3390/s23218673

**Published:** 2023-10-24

**Authors:** Bryan Rodriguez, Prasanna Rangarajan, Xinxiang Zhang, Dinesh Rajan

**Affiliations:** Department of Electrical and Computer Engineering, Lyle School of Engineering, Southern Methodist University, Dallas, TX 75205, USA; prangara@mail.smu.edu (P.R.); xinxiang@mail.smu.edu (X.Z.); rajand@lyle.smu.edu (D.R.)

**Keywords:** 3D scanning, 3D metrology, point cloud processing, Time-of-Flight sensors

## Abstract

One of the challenges of using Time-of-Flight (ToF) sensors for dimensioning objects is that the depth information suffers from issues such as low resolution, self-occlusions, noise, and multipath interference, which distort the shape and size of objects. In this work, we successfully apply a superquadric fitting framework for dimensioning cuboid and cylindrical objects from point cloud data generated using a ToF sensor. Our work demonstrates that an average error of less than 1 cm is possible for a box with the largest dimension of about 30 cm and a cylinder with the largest dimension of about 20 cm that are each placed 1.5 m from a ToF sensor. We also quantify the performance of dimensioning objects using various object orientations, ground plane surfaces, and model fitting methods. For cuboid objects, our results show that the proposed superquadric fitting framework is able to achieve absolute dimensioning errors between 4% and 9% using the bounding technique and between 8% and 15% using the mirroring technique across all tested surfaces. For cylindrical objects, our results show that the proposed superquadric fitting framework is able to achieve absolute dimensioning errors between 2.97% and 6.61% when the object is in a horizontal orientation and between 8.01% and 13.13% when the object is in a vertical orientation using the bounding technique across all tested surfaces.

## 1. Introduction

**Figure 1:**
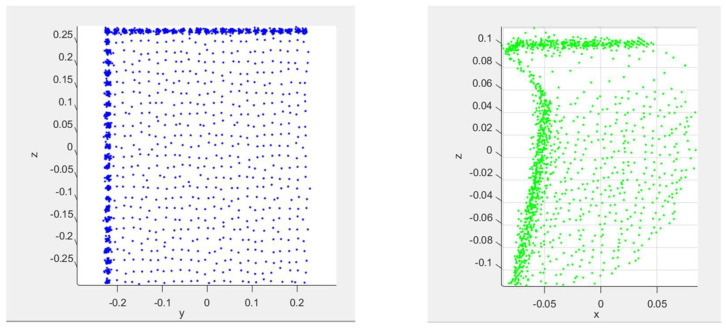
Point cloud of the side profile of a cuboid object without multipath interference (**left**) and with multipath interference (**right**).

This work presents a method for dimensioning cuboid and cylindrical objects from noisy and partially occluded point cloud data that are acquired using a Time-of-Flight (ToF) sensor. Over the last decade, there has been an increase in the types of applications where ToF sensors are used, such as 3D scanning [1,2,3,4,5], drone positioning [6,7,8], robotics [9,10,11], and logistics [12,13,14]. In applications such as metrology and logistics, the ability to accurately determine the dimensions of an object is critical, for example, for part picking, packaging, and estimating shipping costs and storage needs. The quality of the depth information that is provided using modern three-dimensional (3D) sensors depends on the underlying technology. Existing works on dimensioning objects use low-noise, high-resolution 3D sensors such as structured light, stereo vision, and LiDAR to generate depth information [15,16,17]. These 3D sensors each have different tradeoffs and limitations that may not make them suitable for certain applications. For example, stereo vision systems typically have high software complexity, low depth accuracy, weak low-light performance, and limited range [18,19]. As another example, structured lights typically have high material costs, slow response times, and weak bright-light performance [18,19]. Compared to other 3D sensor technologies, ToF sensors provide a low-cost, compact design with low software complexity, fast response time, and good low-light and bright-light performance that can be used in real-time for generating depth information [18,19]. Despite these advantages, ToF sensing suffers from noise artifacts and issues such as multipath interference, which can distort the shape and size of objects in point clouds captured with a ToF sensor. Figure 1 illustrates an example of a point cloud of the side profile of a regular box without and with multipath interference. As shown in Figure 1, multipath interference distorts the profile of the object such that the planar surfaces appear curved. The profile of a cylindrical object also experiences the same type of distortion. The distorted appearance of the object poses a challenge when trying to determine the dimensions of the object. In addition, ToF sensors also suffer from other issues such as low resolution, flying pixels, and self-occlusions, which further makes metrology challenging [20].

**Figure 2:**
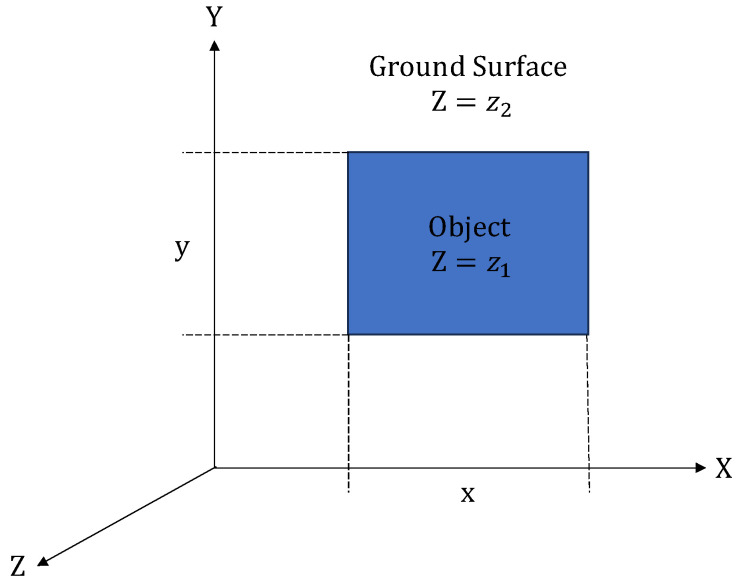
Top-view fronto-parallel configuration of a cuboid object on a ground plane surface.

A common approach to reducing the effect of multipath interference is to place the ToF sensor directly above an object that is placed flat on a ground surface in a top-view fronto-parallel configuration [21,22]. In this configuration, only the top surface of an object is visible to the ToF sensor. An example of a cuboid object in such a top-view fronto-parallel configuration is shown in Figure 2. In this configuration, the *x*- and *y*-dimensions of the object can be readily determined with respect to the *x–y* ground plane. The *z*-dimension can also be readily determined by taking the difference in depth measurements between the top surface of the object and the ground plane surface. In this configuration, since the interface between the object and the ground surface is not visible, the effect of multipath interference between the object and the ground surface is greatly reduced. However, this configuration is only feasible for a limited number of environments and applications.

Using a perspective view of an object proves the most flexibility for applications, but processing point cloud data captured using a perspective view poses challenges due to the presence of self-occlusions, noise, and multipath interference [20]. The amount of noise and multipath interference is scene-specific and depends on the ground surface material an object is resting on and the pose of the object. One approach for dimensioning cuboid objects involves using a plane fitting to identify the various surfaces of a box [23]. This approach typically requires that at least three surfaces of the box are visible to the ToF sensor and uses a RANSAC algorithm [24] on the point cloud of a box for detecting planes that correspond with surfaces of the box. Notably, this approach is limited to cuboid objects and cannot be applied to non-cuboid objects. Our results demonstrate that the number of points that are present on the surface of an object depends on the pose of the object with respect to the ToF sensor. This plane-fitting approach begins to breakdown as the number of points decreases on the surfaces of the object.

In this work, we developed a superquadric fitting framework for dimensioning cuboid and cylindrical objects using point cloud data that are generated with a ToF sensor that has a perspective view of an object. Our approach allows for the dimensioning of objects without requiring a top view of an object and without requiring that three sides of the object be visible to the ToF sensor. Previous works in robotic grasping applications have implemented a type of superquadric fitting to point cloud data for determining the general orientation and size of an object that is to be picked up with a robotic hand [25,26,27,28]. However, these works focused on obtaining rough dimensions for an object for grasping and did not attempt to quantify how accurately the dimensions of the object can be determined in various environments and orientations. Other existing works have employed superquadric fitting to point cloud data for identifying and classifying objects [29,30,31]. The focus of these works is to generally classify objects. These works also do not attempt to quantify how accurately the dimensions of an object can be determined. Further, these works typically rely on point cloud data that are obtained using other types of 3D sensor technologies, which do not suffer from the same types of noise artifacts and issues as a ToF sensor. These works do not suggest or provide any evidence that their approaches can be similarly applied to the same type of noisy point cloud data that are obtained from a ToF sensor. As discussed above, there is a tradeoff between the quality of the point cloud data that can be obtained and the low-cost, compact design of a ToF sensor that can be used in real-time for generating depth information.

Our proposed framework uses a non-linear least squares regression to determine a superquadric shape that best fits the point cloud data for an object while limiting the overgrowth of the superquadric shape. Our experiments show that during the fitting process, the dimensions of the superquadric shape tend to overgrow in the direction where data points are missing for the surfaces of an object due to self-occlusion. This overgrowth leads to significant errors in the dimensions of the object. A previous study by Quispe et al. also noted that superquadric models tend to overgrow during the superquadric fitting process [32]. When a superquadric model overgrows during fitting, the dimensions of the superquadric shape extend beyond the point cloud of the object. This type of overgrowth leads to inaccurate dimension estimates. Quispe et al. used an approach that was inspired by Bohg et al. that involves identifying a symmetry plane within the point cloud of an object and then using projection to artificially generate surfaces that are missing within the point cloud [16,32]. In their approach, the point cloud data that are used are generated using stereovision and RGBD cameras, which do not suffer from the same type of noise issues (e.g., multipath interference) as point cloud data from a ToF sensor. This approach is not suitable for the types of noisy point clouds that are generated using a ToF sensor because multipath interference distorts the point cloud of objects and makes identifying the surfaces of the object more difficult.

This work contributes to the state of the art by (1) developing a framework for dimensioning cuboid and cylindrical objects using enhanced superquadric fitting techniques and noisy point cloud data that are generated with a single ToF sensor. Our results show that a traditional superquadric fitting technique alone are insufficient for accurately determining the dimensions of an object using point cloud data that suffer from issues such as low resolution, self-occlusions, noise, and multipath interference. Our enhanced superquadric fitting techniques include bounding techniques for limiting superquadric overgrowth as well as considerations for the orientation of an object; (2) quantifying the accuracy for dimensioning cuboid and cylindrical objects on various types of ground surfaces using the noisy and partially observed point cloud data from a ToF sensor. The ground surfaces considered in this work include aluminum foil, black posterboard, white posterboard, and black felt. Each of these ground surfaces has different levels of infrared reflectivity; (3) quantifying the accuracy for dimensioning cuboid and cylindrical objects with different rotation angles and orientations with respect to the ToF sensor; and (4) quantifying the accuracy for dimensioning cuboid and cylindrical objects using various techniques for limiting overgrowth when fitting superquadric models. In applications such as logistics, the ability to accurately dimension objects is critical for operations like object grasping, packaging, storing, and transportation [33,34,35,36,37]. The tolerances for dimensioning errors vary from system to system. As these systems are further developed, their tolerances are typically reduced to optimize efficiency, object handling, and packaging [33,34,35,36,37]. As such, it becomes increasingly important to understand and quantify the performance and accuracy of object dimensioning techniques and the various factors that affect their performance. As discussed above, the presence of issues such as low resolution, self-occlusions, noise, and multipath interference all negatively impact and limit the usage of traditional techniques for dimensioning objects using point cloud data. This work contributes to the state of the art by quantifying the performance and accuracy of our proposed framework compared to traditional techniques for dimensioning objects using point cloud data. In addition, this work further contributes by quantifying how various environmental factors, such as ground surface material and object orientation, impact the performance and accuracy of our proposed framework.

This work uses a Texas Instrument TI OPT8241 ToF sensor for its experiments due to its widespread use in research and engineering applications. Since other ToF sensors operate using the same principles, which involve emitting and capturing reflected IR light, our framework for dimensioning cuboid and cylindrical objects using point cloud data from a ToF sensor can therefore also be generally applied to other types of ToF sensors since they all experience the same types of issues such as self-occlusions, noise, and multipath interference [20].

This paper is organized as follows: Section 2 discusses our proposed superquadric fitting framework for dimensioning cuboid and cylindrical objects. Section 3 discusses the experimental setup and the numerical results. Finally, Section 4 provides concluding remarks.

## 2. Methodology

**Figure 3:**
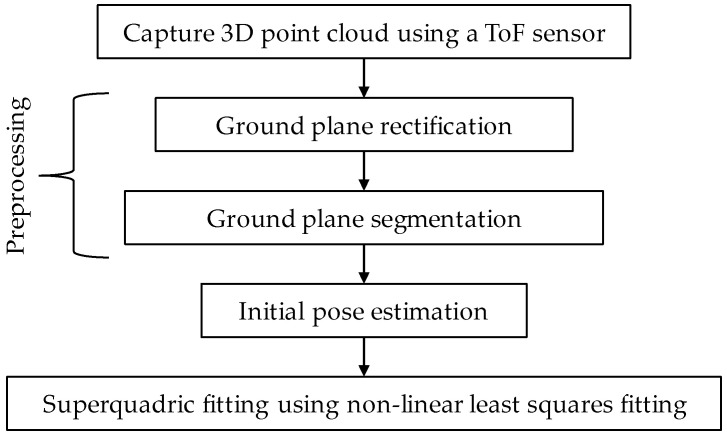
Process for performing parametric fitting for dimensioning of an object.

As discussed above, point cloud data that are obtained from a single ToF sensor typically suffers from issues such as low resolution, self-occlusions, noise, and multipath interference. These issues tend to distort the shape and size of objects, which creates challenges for dimensioning objects using a ToF sensor. In this work, we propose an approach that overcomes these challenges by fitting a parametric model to the point cloud data. Given a set of point cloud data points ($x_{w},\;y_{w},\;z_{w}$) from a ToF sensor, our proposed approach uses non-linear least squares fitting to determine the parametric model that best fits the point cloud data. Our experiments show that directly applying a parametric fit to the point cloud data without any preprocessing results in large estimation errors. To address this issue, in our proposed framework, we preprocess the point cloud data using the following steps: ground plane rectification, ground plane segmentation, and reorienting the point cloud within a new local coordinate system before performing the initial pose estimation. Using this approach, the subsequent parametric fitting shows significantly lower dimensioning errors. Figure 3 provides an overview of our methodology.

The key steps to the parametric fitting for dimensioning methodology are as follows: A ToF sensor is configured to capture a point cloud from a perspective view of an object within a scene. A ground plane rectification process is first performed to compensate for the perspective view of the ToF sensor. Ground plane segmentation is then performed to segment the object from the rest of the scene. An initial pose is then determined for the object, and the point cloud for the object is reoriented such that the object is axis-aligned and centered about a user-defined local origin. The reoriented point cloud is then fed into a superquadric fitting algorithm. As part of the fitting process, we use either a bounding technique or a mirroring technique to limit any overgrowth of the superquadric model. The bounding technique limits superquadric shape overgrowth by applying adaptive upper and lower bounds to the dimensions of the superquadric shape during the fitting process. The mirroring technique limits superquadric shape overgrowth by synthetically generating data points for the surfaces of the object that are missing due to self-occlusion. The mirroring technique generates a more complete point cloud representation of an object that reduces the number of missing surfaces, which would allow the superquadric shape to overgrow during the fitting. The object dimensions can then be obtained based on the determined parameters of the superquadric shape that is fitted to the point cloud.

### 2.1. Ground Plane Rectification

**Figure 4:**
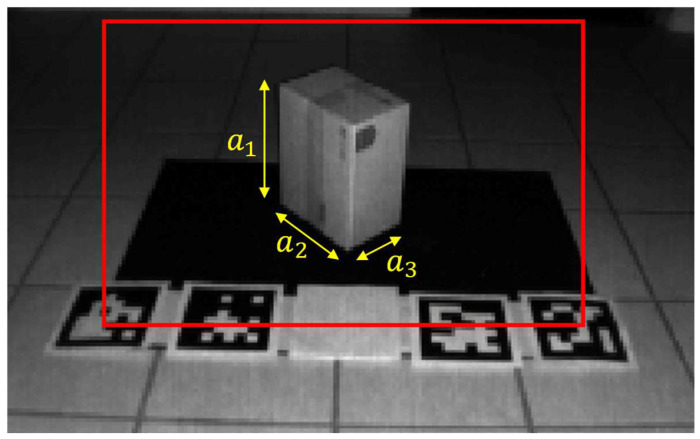
Intensity image of a scene with ArUco markers and a box positioned on a black felt surface. The region of interest (ROI) for our object is represented by the red bounding box.

Figure 4 is an example of an intensity image of a scene with fiducial markers and a box positioned on top of a black felt surface. The dimensions of the box are labeled as $a_{1}$, $a_{2}$, and $a_{3}$. In this example, $a_{1}\;$= 149 mm, $a_{2}$ = 223 mm, and $a_{3}$ = 286 mm. In our experiments, ArUco fiducial markers are initially used to determine the orientation of the ground plane with respect to the ToF sensor. An ArUco marker is a 2D binary-encoded fiduciary marker that can be used for camera pose estimation [38]. ArUco markers were selected due to their widespread use in various computer vision-based applications, such as robotics and automation. This approach does not require a pre-calibrated camera mounting system with respect to the object plane and is more robust and applicable to dynamic settings where a camera is mounted to a movable arm or robot system. Although ArUco markers were used in this work, a similar approach can be implemented using other suitable types of fiducial markers.

**Figure 5:**
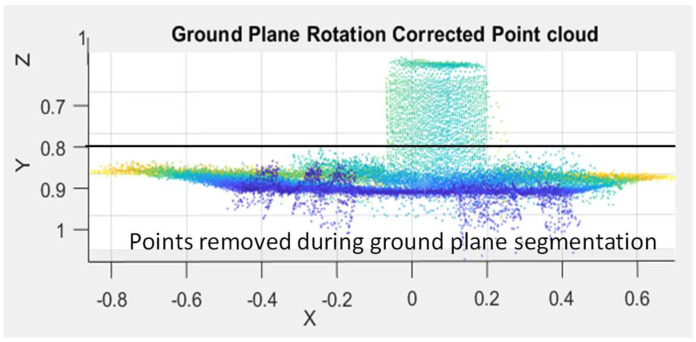
Front view of a point cloud of a scene with a box on a ground plane surface after ground plane correction in meter units. An offset threshold for the ground plane segmentation process is represented by the solid black horizontal line. The color of a data point in the point cloud corresponds with a distance between the ToF sensor and a surface in the scene. Darker colors (e.g., dark blue) represent surfaces closer to the ToF sensor and lighter colors (e.g., yellow) represent surfaces further away from the ToF sensor.

To determine the ground plane orientation, an ArUco marker is placed on the ground plane within the field of view of the ToF sensor. We then capture and process an intensity image of the scene using the OpenCV libraries [39] to detect the presence and orientation of the ArUco marker. The orientation of the ArUco marker provides information about the orientation of the ground plane with respect to the ToF sensor. Once the orientation of the ground plane is determined, the point cloud for the entire scene is then rotated such that the ground plane is aligned with a horizontal *x–y* plane in our coordinate system. Although only one ArUco marker is required to determine the orientation of the ground plane, we use four markers for redundancy. The ArUco markers are also used to identify and crop the region of interest by positioning the object between the outermost ArUco markers. Figure 5 is an example of a point cloud after ground plane rotation correction. As shown in Figure 5, the ground plane in our point cloud data are substantially parallel with the horizontal *x–y* plane after ground plane rotation.

### 2.2. Ground Plane Segmentation

**Figure 6:**
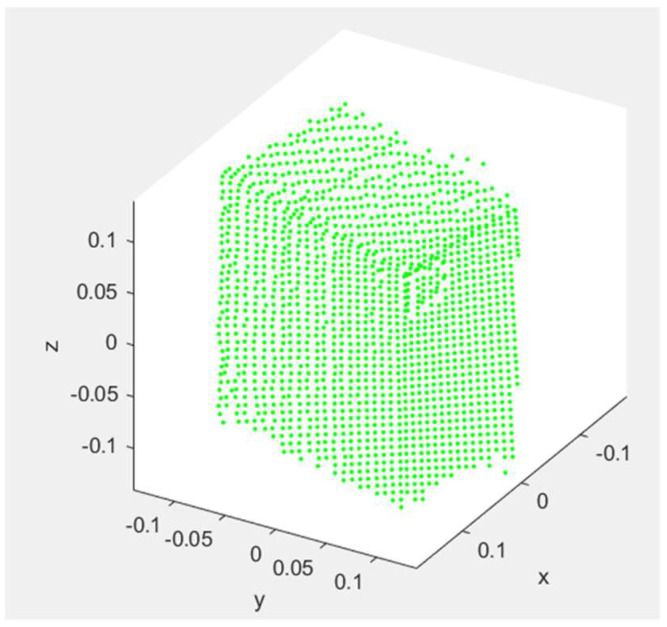
Example of the remaining point cloud data for a box after performing ground plane segmentation in meter units.

Following ground plane rectification, the position of the ground plane is known. Thresholding is then performed using an offset threshold value to segment the object of interest from the ground plane. As shown in Figure 5, the point cloud for the ground plane appears noisy primarily due to multipath interference near the interface between the ground plane and the faces of the object. In this example, additional noise is also caused by the fiducial markers. The noise from the fiducial markers and the multipath interference between the ground plane and the object are removed during segmentation, and the remaining point cloud corresponds to the object of interest. Any residual multipath interference can be reduced by increasing the offset threshold value. The tradeoff for this approach is that increasing the offset threshold value reduces the number of points on the object that are available for the parametric fitting process. Since the offset threshold value is known, this value is added back later as a correction term to one of the dimensions of the object after performing the parametric fitting. Figure 6 shows an example of the remaining point cloud data for a box after performing ground plane segmentation.

### 2.3. Initial Pose Estimation

**Figure 7:**
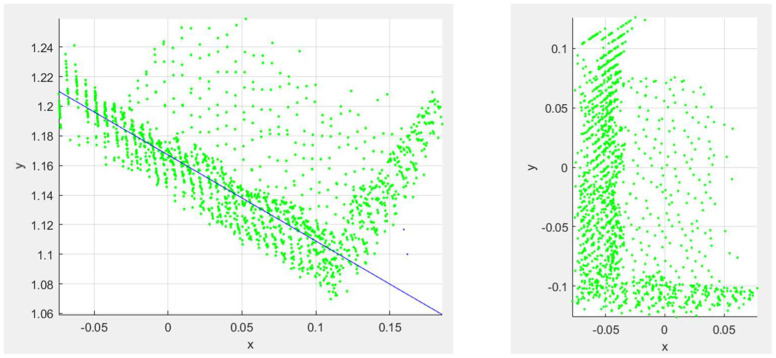
Example of reorienting the point cloud data for a box before (**left**) and after axis alignment (**right**) in meter units. In the left plot, the blue line corresponds with the orientation for an edge of the box that was determined from the RANSAC process.

To determine the initial pose of the object, the remaining point cloud of the object is reoriented such that the object is centered about a user-defined origin and axis aligned. In this work, this reorientation is performed by flattening the point cloud into the direction of the ground plane to form a top-view representation of the object. Figure 7 illustrates an example of the result of the flattening process for the point cloud with respect to the ground plane. By flattening the point cloud in this manner, dense clusters of point clouds will appear, which correspond with the edges of the object. Once the point cloud has been flattened, a RANSAC (“RANdom Sample Consensus”) algorithm [24] is used to identify an edge of the object by fitting a line to one of the edges of the point cloud. The RANSAC process first identifies the dense clusters of points that correspond with the edges of the object and then fits a line to one of these clusters of data points. In the example shown in Figure 7, the line that was determined from the RANSAC process is represented as a solid blue line. Once the orientation of an edge of the object is known, the point cloud is rotated such that the object edges are axis-aligned. First, an angle is determined between the line that was determined from the RANSAC process and either the *x*- or *y*-axis of the coordinate system. Then, the entire point cloud is rotated about the vertical *z*-axis with the determined angle to axis align the point cloud with the *x–y* plane. The axis-aligned point cloud is then shifted such that the center of the point cloud is at the local origin. An example of the result of this process is also shown in Figure 7.

Once the point cloud is centered at the origin, the initial rotation parameters (ϕ, θ, ψ) and translation parameters ($p_{x},\;p_{y},\;p_{z}$) are set to zero. Although the fitting process is capable of solving for non-zero translation and rotation parameters, our experiments showed improvements in terms of accuracy and speed by reorienting our point cloud and initially setting these parameters to zero. The fitting process will later adjust the translation and rotation parameters to best fit the point cloud data. The initial dimension parameters ($a_{1},\;a_{2},\;a_{3}$) of the object are determined based on the difference between the minimum and maximum values of the point cloud along each axis.

### 2.4. Limiting Superquadric Growth

**Figure 8:**
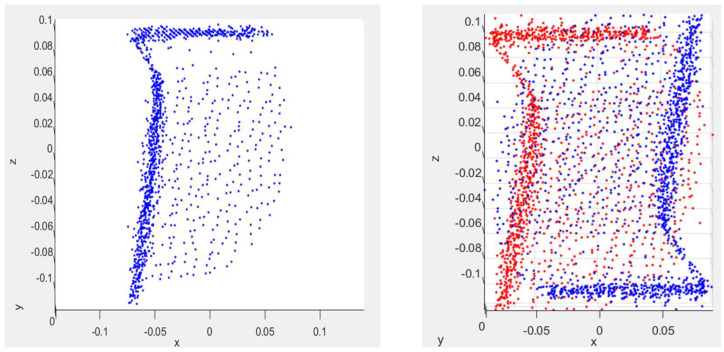
Profile view of a point cloud of a cuboid before (**left**) and after applying the mirroring technique for limiting superquadric overgrowth (**right**) in meter units. In the right plot, the red data points correspond with the initial point cloud of the cuboid and the blue data points correspond with the additional data points that were generated by applying the mirroring technique.

At this point, we can fit a superquadric model to the remaining point cloud data. However, our experiments showed poor performance resulting from overgrowth in the direction where data points are missing for the surfaces of an object due to self-occlusion. In the first set of experiments, the fitting was performed on the axis-aligned point cloud. In these experiments, adaptive upper and lower bounds were used to limit overgrowth on the dimensions of the superquadric model that was generated. In our experiments, tolerances of 5%, 2%, and 1% are applied to the dimensions estimated during the initial pose estimation process to determine the bounds. The upper and lower limits were used because the point cloud of the object is incomplete due to self-occlusions. For example, with cuboid objects, the point cloud data only have points on the surfaces of the box that are visible to the ToF sensor and do not include points that represent the bottom or the backside of the box. In some instances, when a superquadric model is fit to the point cloud with partial data, the superquadric grows beyond the points in the point cloud where the surface of an object is not represented. This occurs because the minima in Equation (2) does not guarantee returning the smallest superquadric model that fits the point cloud data. In a second set of experiments, the fitting process is performed on a mirrored version of the axis-aligned point cloud. The mirrored point cloud is generated by creating a duplicate of the axis-aligned point cloud, flipping it 180° vertically about its centroid and the *x*-axis, and then rotating it 90° about the vertical *z*-axis. The duplicate point cloud is then merged with the original axis-aligned point cloud to form the mirrored version of the point cloud. By using the mirrored point cloud, data points for any non-visible sides of the object are synthetically created, and bounds are no longer necessary to limit any overgrowth in the fitting process. An example of the mirroring technique is shown in Figure 8.

### 2.5. Non-Linear Least Squares Fitting

Our work involves performing a non-linear least squares fit of a superquadric shape to the point cloud of an object to determine the dimensions of the object. A superquadric is a parametric shape that has parameters that describe the size, shape, and pose of the superquadric [40,41]. In this work, a superquadric shape was selected because it can be morphed into a wide range of shapes [40]. By adjusting the shape parameters, the superquadric shape can be morphed into a range of symmetric objects, which include cuboids and cylinders. The implicit form of the superquadric equation that is used in this work is given using the inside–outside function *F*, which is defined as the following:

(1) $$F\left(x_{w},y_{w},z_{w}\right)={\left[{\left(\frac{n_{x}x_{w}+n_{y}y_{w}+n_{z}z_{w}-p_{x}n_{x}-p_{y}n_{y}-p_{z}n_{z}}{a_{1}}\right)}^{\frac{2}{\in_{2}}}\;\;\;\;\;\;\;\;\;\;\;+{\left(\frac{o_{x}x_{w}+o_{y}y_{w}+o_{z}z_{w}-p_{x}o_{x}-p_{y}o_{y}-p_{z}o_{z}}{a_{2}}\right)}^{\frac{2}{\in_{2}}}\right]}^{\frac{\in_{2}}{\in_{1}}}\;\;\;\;\;\;\;\;\;\;+{\left(\frac{a_{x}x_{w}+a_{y}y_{w}+a_{z}z_{w}-p_{x}a_{x}-p_{y}a_{y}-p_{z}a_{z}}{a_{3}}\right)}^{\frac{2}{\in_{1}}}$$

where variables $\left(x_{w},y_{w},z_{w}\right)$ are the data points from the captured point cloud, ($a_{1},a_{2},a_{3}$) are the scaling dimensions along the *x*-, *y*-, and *z*-axis of the superquadric, respectively, ($\in_{1},\in_{2}$) are shape parameters, and ($n_{x},n_{y},n_{z},o_{x},o_{y},o_{z},a_{x},a_{y},a_{z},p_{x},p_{y},p_{z}$) are the twelve parameters of a homogenous transformation matrix that is the result of a rotation and a translation of the world coordinate plane [42,43]. The eleven parameters that define the position and orientation of a superquadric are defined as ʌ = {*a*_1_, *a*_2_, *a*_3_, *∈*_1_, *∈*_2_, *ϕ*, *θ*, *Ψ*, *p_x_*, *p_y_*, *p_z_*} [42,43].

Following our initial pose estimates, the initial rotation parameters $\left(\phi ,\theta ,\Psi \right)$ and translation parameters $\left(p_{x},p_{y},p_{z}\right)$ are set to zero, the initial object dimensions ($a_{1},a_{2},a_{3}$) are determined as the difference between the minimum and maximum values of the point cloud along each axis, and the shape parameters ($\in_{1},\in_{2}$) are initially set to an intermediate value of one. The final values of ʌ are determined using a least squares minimization process. We perform a least squares minimization using the Levenberg–Marquardt algorithm [44] to recover the parameter set ʌ that best fits the *k*th point, ($x_{k},y_{k},z_{k}$), in the point cloud. The following expression describes the minimization process:

(2) $$\underset{k}{\min}\sum_{k=0}^{n}{\left(\sqrt{a_{1}a_{2}a_{3}}\left(F^{\in_{1}}\left(x_{k},y_{k},z_{k};ʌ\right)-1\right)\right)}^{2}$$

where the coefficient $\sqrt{a_{1}a_{2}a_{3}}$ is used to recover the smallest superquadric, and the exponent $\in_{1}$ promotes faster convergence by making the error metric independent of the shape parameters [43].

After determining the parameters ($a_{1},a_{2},a_{3}$) corresponding with the dimensions of the object, the offset threshold value that was used during the ground segmentation step is then added to the vertical dimension of the object to compensate for the portion of the point cloud that was removed during the ground plane segmentation process. Through this process, the full dimensions of the object are recovered.

**Figure 9:**
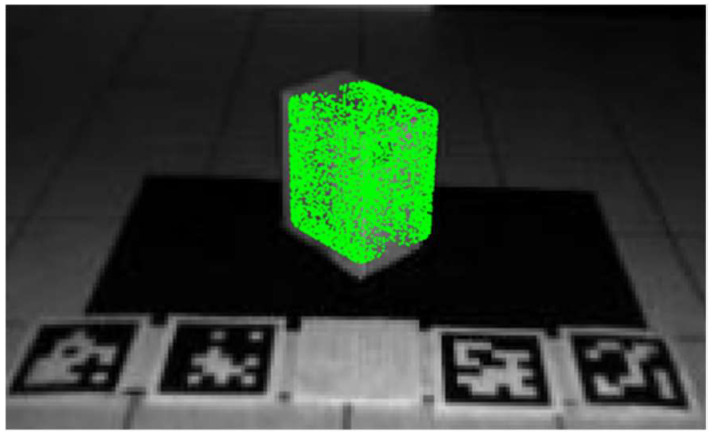
Example of a determined superquadric shape (shown in green) overlaid with a box in an intensity image. The superquadric shape is represented as a series of points corresponding with data points on the surface of the superquadric shape.

Figure 9 is an example of a superquadric shape that is generated based on the parameters determined with the non-linear least squares fitting, which is overlaid with a corresponding object of interest (i.e., a box) within an intensity image.

## 3. Experiments

### 3.1. Hardware Configuration

In our experiments, we use a single ToF sensor, TI OPT8241 [45], to generate depth information for a single object. This sensor is able to output both grayscale intensity images and point clouds. This sensor offers a resolution of 320 × 240 with a horizontal field-of-view of 74.4°. In each experiment, the object is located 1.5 m from the ToF sensor. The ToF sensor is positioned on a tripod with a downward perspective view of between 35 and 45° of the object. The physical dimensions of the boxes are between 122 mm and 365 mm in length. The cylinder has a height of 204 mm and a diameter of 155 mm. In each experiment, an object is placed on different ground plane surfaces that each have different levels of infrared reflectivity and multipath interference. The ground plane surfaces used in our experiment are aluminum foil, black poster board, white poster board, and black felt. For each ground plane surface, the object is rotated between angles of 30 and 75° with respect to the ToF sensor.

For capturing intensity images and point cloud data, we used a Voxel Viewer from Texas Instruments [46]. During the data collection period, an average of 400 frames of intensity images and point cloud data were collected for each experiment configuration. For implementing our framework, we used Matlab R2020b and OpenCV libraries [39,47]. The OpenCV libraries were primarily used for identifying fiducial markers in our ground plane rectification process.

### 3.2. Dimensioning Performance for Cuboid Objects Based on the Ground Plane Surface

Table 1 shows the average of absolute errors for each of the ground surfaces using various dimensioning techniques. As the box is rotated with respect to the ToF sensor, an error for each dimension of the box is computed. The absolute errors for each dimension are then averaged to determine the average of the absolute errors at each rotation angle of the box. The average of absolute errors is the average of the errors across all the rotation angles for the box from 30° to 75°.

Table 1 shows that a traditional approach of fitting an ellipsoid to the point cloud results in large errors compared to the superquadric fit. Errors can be further reduced by using bounding or mirroring techniques to limit the overgrowth of the super-quadric model during the fitting process. Both techniques rely on fitting the superquadric shape after the point cloud is axis-aligned, which reduces the variation in the rotation parameters in the superquadric shape and improves performance. Our results show that the impact of multipath interference from the ground planes having different levels of infrared reflectivity is negligible using either technique.

### 3.3. Dimensioning Performance for a Cuboid Object Based on Object Orientation

**Figure 10:**
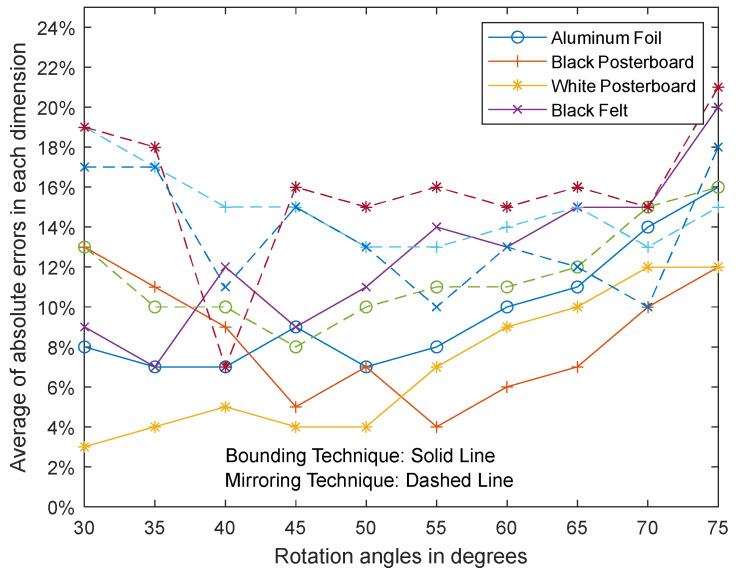
Dimension errors at each rotation angle of the box. Each marker type (i.e., o, +, *, x) corresponds with a ground surface material. Solid or dashed lines are used with the corresponding marker type based on whether the bounding technique or the mirroring technique was applied, respectively.

**Figure 11:**
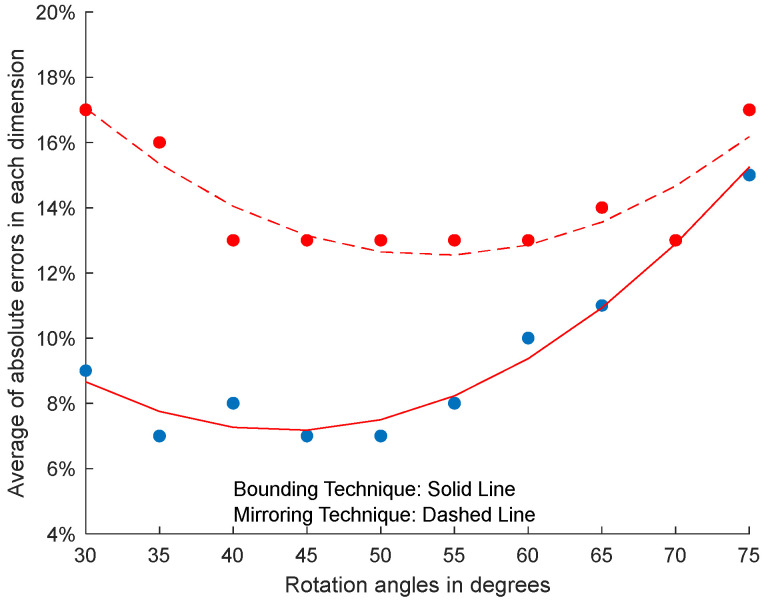
Average dimension errors of the box across all ground plane surfaces. The blue data points correspond with error measurements obtained using the bounding technique. The red data points correspond with error measurements obtained using the mirroring technique.

Figure 10 shows the average of absolute errors for each ground plane surface at each rotation angle of the box using the bounding technique and the mirroring technique. Figure 11 shows the average of absolute errors for all of the ground plane surfaces at each rotation angle of the box with respect to the ToF sensor using the bounding technique and the mirroring technique.

In Figure 10 and Figure 11, the box is initially positioned at an angle of 45° with respect to the ToF sensor such that two side surfaces and the top surface of the object are visible to the ToF sensor. The box is then rotated about the vertical *z*-axis with respect to the ToF sensor to determine the effect of the rotation angle of the box with respect to the ToF sensor. The results from Figure 10 and Figure 11 show that the smallest of the absolute errors for the various ground plane surfaces generally occur when the box is rotated about 45° with respect to the ToF sensor. In this orientation, both vertical faces are most visible to the ToF sensor, which results in more data points on the box surfaces being available for processing. As the box rotates away from 45°, one of the vertical sides of the box becomes less visible, resulting in fewer data points on the surfaces of the box being available for processing. Our results show that the average error increases as the number of data points decreases. In an extreme case, when the box is rotated head-on with the ToF sensor at 0° or 90°, only one vertical surface and the top surface are visible to the ToF sensor, which results in the fewest number of data points on the surfaces of the box. Although the average error increases as the number of data points on the surfaces of the box decreases, our experiments show that superquadric shapes can be used even when only two surfaces of the object are visible to the ToF sensor. While our framework can be applied when only one vertical surface and the top surface are visible to the ToF sensor, our results show that the average error further increases as the number of data points on the surfaces of the box decreases. In particular, the mirroring technique experiences a higher average error compared to the bounding technique since the mirroring technique relies on surface data points for synthetically creating non-visible sides of an object.

### 3.4. Dimensioning Performance for a Cuboid Object Using Bounding Technique

Table 2 shows the average of absolute errors for each of the ground plane surfaces with a box rotation of 45° with respect to the ToF sensor using the bounding technique and the mirroring technique for fitting a superquadric shape to the point cloud data. Our results show that the bounding technique can provide lower dimensioning errors compared to the mirroring technique. In our experiments, we observed that the mirroring technique tends to result in underfitting the superquadric shape to the point cloud data, which results in larger dimensioning errors.

### 3.5. Dimensioning Performance for a Cylindrical Object Using Bounding Technique

**Figure 12:**
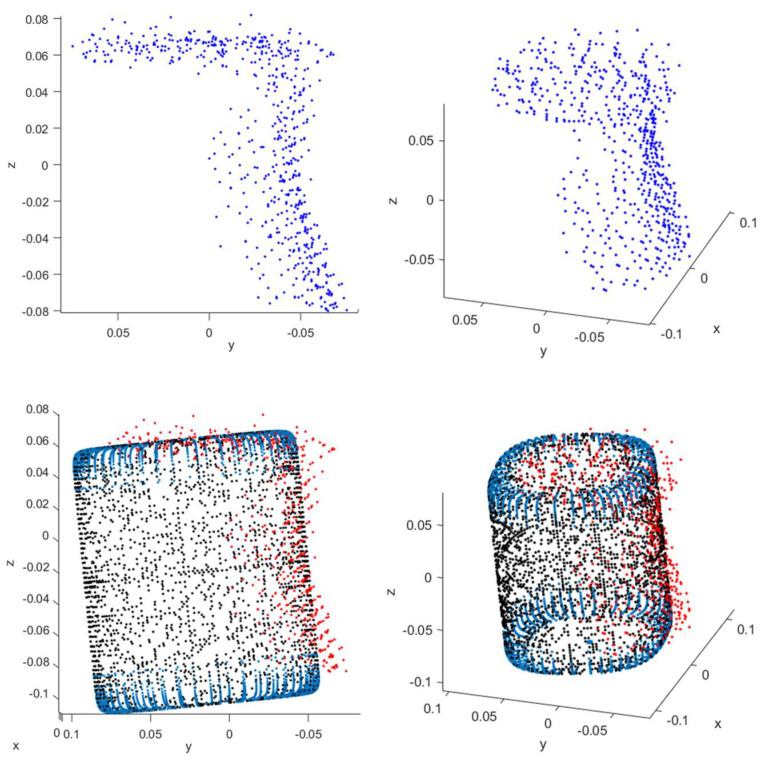
Profile view (**top left**) and perspective view (**top right**) of a point cloud for a cylindrical object and profile view of superquadric fit (**bottom left**) and perspective view of superquadric fit (**bottom right**) to the point cloud for the cylindrical object in meter units. In the bottom plots, the red data points correspond with the point cloud for the cylindrical object and the black and blue data points corresponds with data points on the surface of the superquadric shape that was determined from the superquadric fitting process.

Based on the findings from the cuboid object experiments, we conducted similar experiments on a cylindrical object using the bounding technique since it provided better performance than the mirroring technique. Table 3 shows the average of absolute errors for each of the ground surfaces for different orientations of a cylinder using the bounding technique. As the cylinder is rotated with respect to the ToF sensor, an error for each dimension of the cylinder is computed. The absolute errors for each dimension are then averaged to determine the average of the absolute errors at each rotation angle of the cylinder. Table 3 shows that dimensioning the cylindrical object in the vertical orientation resulted in larger dimensioning errors compared to when the cylindrical object was in the horizontal orientation. In both orientations, our experiments showed an increase in the amount of missing data for cylindrical objects compared to cuboid objects due to the curved surfaces of the cylindrical object. These curved surfaces deflected more infrared light away from the ToF sensor, which resulted in less surface data being collected by the ToF sensor. Our experiments also showed that when the cylindrical object is in the horizontal orientation, the proximity of the ground plane to the curved surface of the cylinder reduces the amount of surface data that is lost compared to when the cylindrical object is in the vertical orientation. Figure 12 illustrates an example of the point cloud data for a cylindrical object and the corresponding superquadric fit to the point cloud data.

## 4. Conclusions

In this work, we developed a framework that can be used for dimensioning cuboid and cylindrical objects from point cloud data generated using a ToF sensor despite the presence of noise artifacts and issues such as low resolution, self-occlusions, noise, and multipath interference. This work also quantifies the impact on the accuracy of dimensioning objects based on various model fitting techniques, the pose of an object, the shape of an object, and the ground surface material under an object.

Our results show that the performance of dimensioning a cuboid object increases when more surfaces and surface areas of the object are visible to the ToF sensor. Conversely, the performance of dimensioning a cuboid object decreases when fewer surfaces and surface areas are visible to the ToF sensor. In addition, the performance of dimensioning a cylinder object increases when the object is in a horizontal configuration as opposed to a vertical configuration. Our results also showed that dimensioning performance improves when a bounding technique is employed in conjunction with the parametric fitting process to reduce overgrowth of the superquadric shape. Notably, the bound-based approach provides better performance compared to a mirroring-based approach that synthetically creates missing point cloud information for an object.

This work can be extended to examine the use of multiple ToF sensors to further improve dimensioning accuracy. Future works may also extend the ability of parametric fitting to dimension more complex shapes with non-convex surfaces.

## Figures and Tables

**Table 1:** Dimension errors for a box using various surfaces and fitting techniques.

Method	Aluminum Foil	Black Posterboard	White Posterboard	Black Felt
Ellipsoid fit	99%	115%	79%	102%
Superquadric fit— no bounds or axis alignment	37%	228%	40%	33%
Superquadric fit— 1% bounds with axis alignment	13%	10%	11%	13%
Superquadric fit— Mirroring	11%	15%	17%	13%

**Table 2:** Dimension errors for various fitting techniques of a box rotation angle of 45°.

Method	Aluminum Foil	Black Posterboard	White Posterboard	Black Felt
Superquadric fitting— 1% Bounds w/axis alignment	9%	5%	4%	9%
Superquadric fitting— Mirroring	8%	15%	16%	15%

**Table 3:** Dimension errors for a cylinder using the bounding technique.

Cylinder Orientation	Aluminum Foil	Black Posterboard	White Posterboard	Black Felt
Horizontal	6.61%	6.35%	2.97%	6.29%
Vertical	8.01%	12.75%	12.87%	13.13%

## Data Availability

Not applicable.
